# Framework restoration on Tang Dynasty garden as a multiple-histories environment: Regions, ecology, architecture, and human behavior

**DOI:** 10.1016/j.heliyon.2024.e35190

**Published:** 2024-07-26

**Authors:** Biyu Huang, Zhenwei Zhang, Wende Chen

**Affiliations:** a School of Architecture and Civil Engineering, Xiamen Institute of Technology, Xiamen, 361000, China; b School of Civil Engineering and Architecture, Xiamen University of Technology, Xiamen, 361021, China

**Keywords:** Tang dynasty garden, Feature framework, Restoration, Risk factors, Protective factors

## Abstract

From a multiple-histories perspective, this paper attempts to restore the feature framework of Tang Dynasty gardens by describing the environment panorama of that era. Tang Dynasty gardens have their own unique and complex environment features, which is crucial for understanding Chinese classical gardens. This research developed a massive text mining method of historical-document to detect all garden-related elements in Tang poetry. By sorting out these elements, it has been restored the original appearance of Tang Dynasty gardens and summarized its feature framework. The resulting model included 199 factors, 173 of which are correlating with gardens. Among the 173 factors, 129 are risk factors and 44 are protective factors. This paper restores the feature framework of Tang Dynasty gardens from the following four points, namely regions, ecology, architecture and human behavior. Understanding the Tang Dynasty gardens would help understand the development context of Chinese classical gardens, and should provide new paths for contemporary environment creation.

## Highlights

• This paper conducts a quantitative study based on Tang poetry.
• Large sample size enhances credibility of this study.
• Study restores the feature framework of Tang gardens from four aspects.
• The research findings are illustrated through case study.

## 1 Introduction

### 1.1 Background

Purpose of this study is to explore the feature framework of Tang Dynasty gardens, and to restore it panoramically. Although there are few relics and many documents, the overall environmental features of Tang Dynasty gardens are vague and unclear, so it is necessary to search for various fragments in order to recover their feature framework. Unlike the well-known gardens of the Ming and Qing Dynasties, the gardens of Tang Dynasty are still a relatively unfamiliar historic environment, both to the public and scholars. Ming and Qing gardens, represented by Suzhou gardens, are well known to the world because of their distinctive style. However, it would be a big mistake to think that the gardens of the Tang Dynasty were the same as them. Research on the distribution and scale evolution of Suzhou gardens shows that compared to the gardens of Ming and Qing Dynasties, gardens of Tang Dynasty were larger in scale, had fewer buildings, were more natural, and were slightly rougher [1]. Anyway, people still lack a systematic understanding of how gardens of Tang Dynasty had existed. This is attributed to the following two reasons. First, there are too few heritages and they have been rebuilt over the years [2]. Second, although there are many documentary records, they are usually not detailed and in-depth enough [3]. Occasionally, archaeological research has touched on this area, but the information provided is very limited. Exploring the feature framework of gardens in that era has become a very meaningful and challenging topic, which is indeed the most important research objective of this paper. Another objective of this paper is to understand the daily lifestyle of Tang Dynasty through the study of gardens.

Comprehending environment of Chinese classical gardens including Tang Dynasty gardens, necessitates a multiple-histories perspective, encompassing not only expertise in environmental science, ecology, agronomy, and forestry but also an imperative framework rooted in history, socioeconomic factors, historical geography, literature, aesthetics, and other pertinent disciplines [4]. Through analysis of Tang poetry, people can interpret the above-mentioned comprehensive information, including ecology and socio-culture. As famous environmental samples, Chinese classical gardens have an outstanding reputation. Adhering to the Chinese Taoist concept of "harmony between heaven and man”, Chinese classical garden is generally recognized as a model of sustainable ecological environment [5]. It fully considers the influence of natural factors and human activities, and realizes the harmonious coexistence between man and nature [6]. Chinese classical garden has played an important role in social life throughout the ages, providing people with places for recreation and cultural activities [7]. Gardens of Tang Dynasty occupy a key position in Chinese classical gardens. As a milestone in the history of Chinese classical garden, Tang Dynasty gardens inherited the excellent tradition since the Qin and Han dynasties, laid the foundation for the prosperity of garden in the Ming and Qing dynasties [8]. At the same time, Tang Dynasty gardens were pioneering in terms of gardening techniques and artistic styles. Thanks to the cultural prosperity at that time, poetry and painting art were introduced into the image of garden construction [9]. The active participation of literati and officials promoted the vigorous rise of literati garden [10]. Apparently, due to the vast territory and open culture of the Tang Dynasty, the gardens of the Tang Dynasty absorbed the essence of foreign culture and enriched their own artistic connotation [11]. In short, the Tang Dynasty gardens fully absorbed the achievements of architecture, landscape, poetry and painting at that time, creating a harmonious but different ecological environment. Throughout the environmental history in the world, Tang Dynasty gardens have provided many distinctive and beneficial ecological and socio-culture values. First of all, gardens in the Tang Dynasty promoted the harmonious coexistence between man and nature. Secondly, it emphasizes the harmonious coexistence of nature and architecture [12]. Thirdly, the Tang Dynasty gardens paid attention to plant landscaping, made a large amount of use of plant materials, which provided a reference for the greening and ecological construction of modern cities [13]. finally, it was thoughtfully committed to sustainable development, which was conducive to the regeneration and reconstruction of the environment [14].

Compared with previous studies, this study has some differences and innovations. This article developed a method to mine a large number of documents, using it to retrieve elements related to gardens in Tang poetry and systematically organize them. Based on environmental feature, the author reconstructed the framework of Tang dynasty gardens. It fills a knowledge gap regarding Tang dynasty gardens, offering fresh perspectives and methods for studying the characteristics of Tang dynasty gardens. Furthermore, the selection of statistical samples is innovative. The text of each sample contains 40 words, which makes the application of this statistical method more rigorous and reliable. This study applies quantitative methods to find correlation factors and classify them. It employed logistic regression to analyze data from a comprehensive collection of Tang poems consisting of 13,100 poems with five-character and eight-line poem. The objective was to detect all elements related to gardens in the context of Tang poetry. The final model contained 199 factors, of which 173 were garden-related, including 129 risk factors and 44 protective factors. After sorting and classifying these factors, the study summarized the four aspects of the Tang Dynasty gardens: regions, ecology, architecture and human behavior. Therefore, this article will conduct a literature review from these four aspects.

### 1.2 About regions

**Fig. 1:**
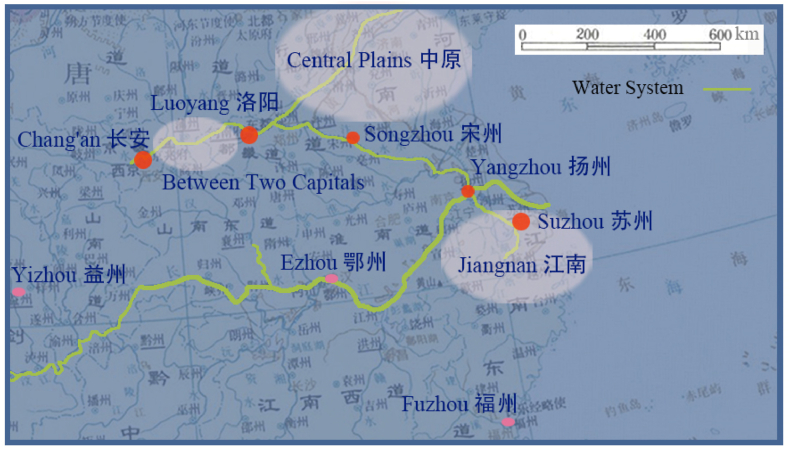
Frequently mentioned regions where gardens flourished during Tang Dynasty.

In Tang Dynasty, regional distribution of gardens was closely related to economic development and population distribution. Exploration in this area helps people understand the overall regional attributes of Tang gardens. Wherever economy was prosperous, there were many gardens. Most scholars agree with this view. Excavation of the canal by Emperor Yang was a major event. It connected the Yangtze River and the Yellow River, and also connected the south and the north. Through it, materials from Jiangnan were continuously transported to Chang'an and Luoyang to meet the needs of expanding population. This measure directly contributed to the great economic development of the southern region. As show in Fig. 1, the economic centers of the Tang Dynasty mainly included the following three areas: first, two capitals and the cities between the two capitals [15], second, Central Plains, which were traditional wealthy farming area, and third, the emerging economic area Jiangnan [16]. Fenfang Chen studied the distribution relationship between the population and gardens in China's past dynasties, and found that the distribution of gardens in Tang Dynasty was roughly consistent with the population distribution [17]. The study also showed that more gardens had appeared in Sichuan, Hubei and Fujian. As Capital, there was a complete garden system combining with landscape and artificial work in Chang'an. According to functions, they could be divided into five categories: royal gardens, government gardens, private gardens, temple gardens and public gardens [18]. In the second capital Luoyang, officials, retired officials and wealthy people built a large number of wonderful gardens. Different from the formal feeling of Chang'an, Luoyang was a more charming city. Everyone loved its relaxed and casual vibe [19]. The famous poet Bai Juyi lived here after his retirement, although he also had a garden in Chang'an [20]. Of course, he also built the Lushan Cottage in Jiangxi, which belongs to Jiangnan [21]. More than 300 km east from Luoyang was Songzhou. There was a long-standing Liang Garden here, which was the residence of King Liang during Western Han Dynasty. Till Tang Dynasty, although the Liang Garden was somewhat dilapidated, it particularly attracted poets' search for historic sites. At least 70 poets during that era wrote 188 poems about this quaint garden [22]. The Great Canals left over from the Sui Dynasty brought unprecedented prosperity to Yangzhou City, because it was an important node of the canals. During Tang Dynasty, Yangzhou City was a city on the water [23]. “Most of houses are around gardens, and there are more boats than gardens [24].” Suzhou, 200 km southeast of Yangzhou, was an ancient historic city. In Tang Dynasty, it was densely populated with flourishing gardens [25]. As mayor of Suzhou, Bai Juyi began to use Taihu stones to build his own garden [26]. At the same time, he also built the Qili Mountain Pond, a landscape channel that connected Huqiu and Suzhou City [27]. On the southwest of the empire, Sichuan had always been the strategic guarantee of the country since Qin Dynasty. On the edge of Guihu Lake in Zizhou, officials built the South Pavilion as a public garden next to the post station [28]. Furthermore, a multitude of exquisite gardens had emerged in the remote and sweltering southern regions. The southward shift of the economic center is further supported by this phenomenon, serving as another credible evidence [17,29].

### 1.3 About ecology

Ecological construction of gardens in the Tang Dynasty fully considered aspects of life, production, and aesthetics. Its diverse and unique ecological features are of great significance. Thanks to the warm climate [30], gardens in Tang Dynasty had a harmonious, beautiful and elegant ecology. The prosperity of art greatly promoted the development of gardening concepts. Wang Wei, a poet and painter, built his Wangchuan Villa with a fusion concept of painting and poetry. This made it not only focused on visual effects, but also on the expression of artistic conception [31,32]. This kind gardens can satisfy human beings' immersive experience, and it pursues the harmony of ecology with the senses [33]. Bai Juyi built four gardens in his life. He wrote many poems to elaborate on his gardening principles. He believed that the site must be located in a place with beautiful scenery, and the garden should be able to enhance the beauty of the original environment [34]. Significantly, he became obsessed with the art of stone, especially Taihu Stone [35,36]. This led to the stone fashion in later generations [37]. Influenced by poetry and painting theory, the plant configuration of Tang Dynasty gardens emphasized naturalness and elegance. Chinese classical literature has a tradition of expressing moods with plants. As expected, plants are used to set off the atmosphere [38]. Common ornamental plants included pine, bamboo, banana, lotus, plum blossom, willow, peony, rose and so on [13]. Luyan Wang et al. [39] studied the plants of the Daming Palace in the Tang Dynasty and found that in addition to common plants, there were also locust trees, magnolias, sycamores, osmanthus trees and cypress trees. The harem of the Daming Palace planted pomegranates, pears, peaches, apricots. Some exotic species was also introduced, such as cherries and grapes. In addition to this, heather, reeds and wild rice were occasionally mentioned. Relying on abundant water systems, many cities in Tang Dynasty were vast garden cities. Various gardens also connected their internal water systems to the huge water systems of city. For example, Chang'an in Tang Dynasty had 5 artificial water systems [40], Luoyang had 4 artificial water systems [41], and Yangzhou was even more magnificent, which was a complete water city [42]. Similarly, Hangzhou City was perfectly integrated with Qiantang Lake (today's West Lake).

### 1.4 About architecture

Architecture of the Tang Dynasty was distinguished by its magnificent grandeur and seamless integration with nature, thanks to the highly developed economy and advanced technology. As a pivotal element in Tang Dynasty gardens, architecture played an indispensable role in creating spatial aesthetics. Exploration in this aspect is conducive to understanding the physical characteristics of Tang gardens. Currently, study on Tang architecture mainly comes from the following three aspects: first, existing buildings, second, archaeological sites, and third, paintings of Tang Dynasty. Wooden buildings are the mainstream of traditional Chinese architecture, but due to moisture, fire or war, it is difficult for them to be preserved for more than 200 years. Since Liang Sicheng discovered Foguang Temple in 1937 [43], there have been a total of five wooden buildings identified as being from the Tang Dynasty, three of which are relatively complete, and two of which are incomplete [44]. Compared with Ming and Qing dynasties, the wooden buildings of the Tang Dynasty have larger brackets due to their stress-bearing features. This results in the brackets and roof being half the height of the building [45]. Thence, they appear to be beautifully proportioned with grand cornices [46]. Another type of Tang Dynasty building that has survived more is the masonry structure, which usually exists in the form of Buddhist pagodas [47]. Number of these pagodas is at least 130. The Qianxun Pagoda of Chongsheng Temple in Dali could represent the style of pagodas in Tang Dynasty, which has a square plane, a single-tube structure, and a dense eaves style tower body [48]. On the other hand, multiple archaeological site excavations have shown that the layout of Tang Dynasty buildings mostly conform to the "Yingzao Fashi” regulations [49]. Archeology of the Daming Palace, the most important palace of the Tang Dynasty, shows that compared with the Ming and Qing Dynasties, the architectural complexes of Tang Dynasty were grand in scale, with sparse layouts, huge squares, and larger building sizes [50,51]. The ruins of Shangyang Palace in Luoyang provide some valuable evidences. First, the waterfront was treated with natural transitions of soft pebbles. Second, the Diao Dian building spanned both sides of the bank. Third, the rockery was a kind of auxiliary landscape, which was relatively rough and covered with stone and earth. These treatments can still be seen in Japanese gardens today, but they are indeed different from China's Ming and Qing gardens [52]. Of course, the existing paintings of the Tang Dynasty also show us the brilliant architectural art, especially the murals of the Mogao Grottoes in Dunhuang. Through them, people not only glimpse the magnificence of Tang Dynasty architecture, but also see the diversity, such as various forms of roofs, brackets and platform bases [53,54].

### 1.5 About human behavior

Tang people embraced gardens as an integral part of their daily existence, seamlessly intertwining their lives with these horticultural spaces. Comprehension of the lifestyle in Tang Dynasty gardens could be achieved through the study of human behavior. Qujiang Pool was a representative of open gardens. It was located in the southeast corner of Chang'an and was famous for its majestic Dayan Pagoda. During all important festivals, there was the most important social place for capital people. The reason why the festivals were so lively is that His Majesty the Emperor would visit as scheduled. Especially during the Shangsi Festival, the emperor would visit the Ziyun Tower to enjoy himself with the people [55,56]. The poet Fu Zai once recorded the grand activities of the Shangsi Festival, which included various theatrical performances and dragon boat races [57]. The Banquet for newly Jinshi, who would immediately become senior officials of the empire, was one of the most famous events here. Celebrations were all held in Qujiang, which attracted the attention of people from all over Chang'an [58]. Ci'en Temple located in Qujiang was the most famous temple at that time. Inside it stood the towering Dayan Pagoda. A must-do activity for new Jinshi was to write their names under the Dayan Pagoda [59]. In addition to Qujiang Garden, famous public gardens in Tang Dynasty included Leyouyuan, Kunming Pool, Meibei Lake, Peach Blossom Pond, Fanchuan, etc. People enjoyed them for spring outings, mountain climbing, outings, flower viewing, and boating [60]. On the other hand, as an official garden, Yanhuachi garden in Chongzhou was famous for Du Fu's poems [61]. Near chang'an, the Wangchuan Villa built by Wang Wei was a representative of private gardens. Its various scenic spots were famous for their elegance and tranquility. The poet lived here in seclusion, practicing Zen and chanting sutras. In this garden, he could become a farmer, a fisherman, or a forest manager like Zhuangzi [31]. More interestingly, in Fuzhou, far to the southeast of the empire, Cui Ciyuan built gardens for viewing polo, which was an aristocratic fashion at the time [62]. In addition to daytime activities, Tang Dynasty gardens also had rich and colorful nighttime activities, where owners socialized, feasted, played musical instruments, admired flowers, worshipped Buddha, and so on [63].

## 2 Methods

### 2.1 Correlation of Chinese characters between "garden” and others

This research requires a type of methods to process huge amounts of information sources, and this type is text mining. The text mining can be used not only in the study of contemporary issues, but also in the mining of historical documents [64]. To restore the feature framework of Tang Dynasty gardens, a huge amount of information sources is needed, otherwise it is difficult to obtain universal characteristics. The larger the information base, the more information can be obtained, and the more accurate universal features can be extracted. Logistic regression as a text mining method is indeed often used in medical research. The medical research often uses it to mine huge amounts of information and extract risk factors and protective factors. In the medical research, if a certain factor appears, causing the probability of disease to be higher than normal level, this factor is called a risk factor; on contrary, if a certain factor appears, causing the probability of disease to be lower than normal level, this factor is called a protective factor [65]. Of course, this study is not a medical study, but we use the terms risk factors and protective factors in accordance with academic convention.

Logistic regression is a widely used statistical method in various fields, including natural language processing. In the context of studying the correlation between Chinese characters, logistic regression provides a quantitative approach to express mathematical relationships between dependent and independent variables. Chinese text, especially classical literature like that from the Tang Dynasty, often exhibits nonlinear correlations due to the interdependence of characters. Logistic regression is well-suited for analyzing such complex relationships. Compared with conventional linear regression methods, the advantage of logistic regression is that the independent variable could be continuous or discrete, which does not have to satisfy the normal distribution. As an important method, linear regression is also used to describe the relationship between independent variables and dependent variables. However, since it requires accurate data, and the dependent variable must be as linear as possible, the use of linear regression methods in practical scenes have encountered many difficulties, which should result in inaccurate final prediction results [66]. Although logistic regression is essentially a generalized linear model like linear regression, it is often used for data classification rather than processing regression problems [67]. Therefore, it has been chosen as the primary analytical tool in this study. Moreover, some algorithms that rely on neural networks are also popular in Large Language Model. This method is suitable for exploring the structural relationship of language, but it is not good at focusing on a keyword.

In the analysis using logistic regression, this research has identified a total of 199 factors that are related to the occurrence of the Chinese character "garden.” These factors can be categorized into two groups: risk factors and protective factors. Risk factors are those that increase the likelihood of encountering "garden” within Chinese texts. On the other hand, protective factors decrease this likelihood of encountering it. It's important to note that when discussing risk and protective factors in medical research, they refer to variables that either elevate or decrease disease occurrence beyond normal levels. This concept has been adapted here to understand patterns within literary data during the Tang Dynasty. By examining these correlations between "garden” and other Chinese characters through logistic regression analysis, study aim to unveil aspects of gardens during this historical period. Overall, logistic regression serves as an effective tool for quantitatively studying nonlinear correlations.

### 2.2 Data source and process

**Fig. 2:**
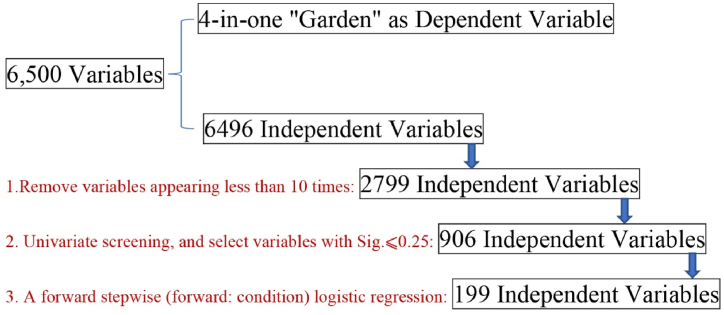
Data processing.

In this article, it has been dedicated extensive to compile a comprehensive database that encompasses all five-character and eight-line poems within the realm of Complete Tang Poems, as shown in Appendix 5. Complete Tang Poems initiated by Emperor Kangxi, compiled during the reign of Emperor Kangxi in the Qing Dynasty. It is recognized as the best and most comprehensive collection of all Tang poetry samples to date. In this version, many ancient poetry professionals had participated in this work. This compilation activity based on the standards of national cultural projects has minimized its error rate [24]. In order to ensure the reliability of the data, it adopts the combination of network mining and manual inspection. From this source, study extracted all 13,100 five-character and eight-line poems to serve as samples for the research. In order to analyze these samples effectively, study selected 6500 Chinese characters as variables. Among them, 3500 were chosen from first-level Chinese character lists while another 3000 were derived from second-level Chinese character lists. By representing each sample with either a "1″ or "0″ depends on whether it shared identical Chinese characters with the variable or not respectively. In this way, research create a huge binary table that allows us to quantify the relationship between different Chinese character variables. By assembling poems with equal character into the analysis process, study has enhanced the consistency form of samples. This meticulous approach ensures that the findings are based on consistent criteria across all analyzed works within Complete Tang Poems. The data processing journey involved transforming unrefined material into ultimate outcome through rigorous steps. Fig. 2 illustrates this process comprehensively by showcasing how raw data of Complete Tang Poems was refined systematically. The research has produced an extensive Excel spreadsheet with 13,100 rows and 6500 columns, comprising a total of 85,150,000 data points. Among the 6500 Chinese character variables, four Chinese characters were merged into the dependent variable, namely "Yuan 园, You 囿, Yuan 苑, Pu 圃", and the other 6496 characters were set to independent variables. In these independent variables, Chinese characters appearing less than 10 times have been eliminated, resulting in a reduction of variables from 6500 to 2799.

Subsequently, the remaining 2799 Chinese characters were treated as independent variables for further investigation. As showed in Eq. (1), symbols for the formula refer to the following: α is the regression intercept parameter; $P_{i}$ represents the probability of an event occurring; and $\beta_{i}$ denotes the regression coefficient of $x_{i}$ (i = 1, 2, …, n), where $x_{i}$ refers to the independent variable. IBM SPSS Statistics 19 was used for calculations, employing the following formula:

(Eq.1) $$P_{i}=F\left[\alpha +\sum_{J=1}^{m}\beta_{i}x_{i}\right]=\frac{1}{1+\exp\left[-\alpha +\sum_{J=1}^{m}\beta_{i}x_{i}\right]}$$

Firstly, study performed a univariate screening process. This involved conducting 2799 logistic regressions. During this process, variables with a significance level higher than 0.25 were excluded, while those with a significance level of 0.25 or lower were selected. As a result, a total of 906 independent variables was obtained. Given the sample size of 13,100, the ratio between samples and independent variables was suitable for logistic regression analysis. It is worth noting that selecting variables with significance values within the range of 0.25 in single factor screening is considered to be a conventional practice aimed at preventing the omission of crucial factors. Next, study employed forward step-by-step multivariate logistic regression to identify correlated variables using the forward condition method. Probability threshold for entry was set at 0.05. Statistical significance was determined when the Sig. Value was below 0.05. Following extensive calculations, a model comprising of 199 predictors was ultimately obtained, indicating that only a small proportion of the total 6500 characters are associated with the term “garden”. Please refer to Appendix 1 for a comprehensive breakdown of the calculation process.

## 3 Results

**Fig. 3:**
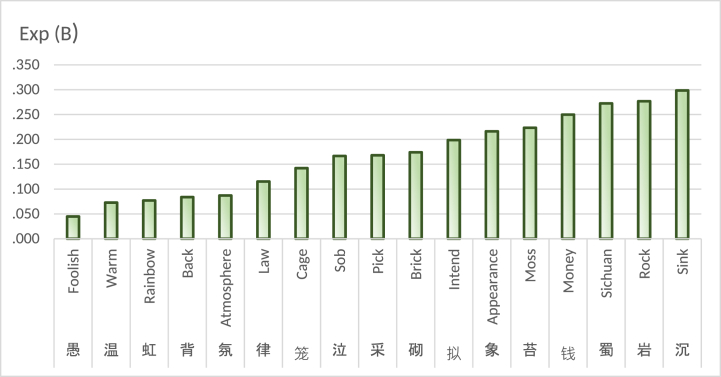
Important Protective factors (Exp (B) < 0.3).

The final model yielded 199 factors. Generally, statistics uses Significance values to determine statistical significance, and Exp (B) values to identify risk or protective factors. The results classify factors as "risk factors” if their Exp (B) value is greater than 1, and as "protective factors” if it is less than 1. The presence of risk factors raises the chances of the dependent variable occurring, while protective factors lower its likelihood. Table 1 shows the complete results: The final model obtained by logistic regression analysis includes 199 independent variables. In results, Significance value of 173 variables was less than or equal to 0.05, and the Significance value of the remaining 26 variables exceeded the threshold of 0.05. Furthermore, the result successfully passed the Hosmer-Lemeshow test, and the obtained Significance value of 0.416 exceeds the predetermined threshold of 0.05. In final model, the Nagelkerke's R2 yielded value of 0.348. The Cox & Snell's R2 yielded value of 0.121.Appendix 2refers to complete results. Appendix 3 refers to the final model, and Appendix 4 is for the last binary-table used in this study, and Appendix 5 is for the original and complete binary-table. Out of the 173 correlation factors, 129 are identified as risk factors while 44 are classified as protective factors. Table 2 presents a list of important risk factors with Exp (B) values exceeding 3, whereas Fig. 3 is a list of important protective factors.

**Table 1:** 199 Variables in the equation.

	B	S.E,	Wals	Sig.	Exp (B)		B	S.E,	Wals	Sig.	Exp (B)
小	0.906	0.174	27.245	0.000	2.474	前	−0.434	0.179	5.860	0.015	0.648
山	−0.232	0.100	5.311	0.021	0.793	洛	1.120	0.209	28.784	0.000	3.066
亿	2.490	0.930	7.172	0.007	12.060	给	1.615	0.598	7.298	0.007	5.025
及	0.639	0.261	5.994	0.014	1.895	袁	−19.785	4723.541	0.000	0.997	0.000
门	0.317	0.130	5.981	0.014	1.374	莺	0.573	0.253	5.132	0.023	1.774
已	0.430	0.145	8.804	0.003	1.538	根	0.987	0.287	11.854	0.001	2.682
丰	1.152	0.436	6.980	0.008	3.165	原	0.609	0.245	6.193	0.013	1.840
井	0.616	0.288	4.581	0.032	1.851	鸭	2.359	0.674	12.238	0.000	10.581
云	−0.320	0.118	7.338	0.007	0.726	钱	−1.387	0.593	5.477	0.019	0.250
午	−18.592	4900.363	0.000	0.997	0.000	租	2.419	0.916	6.978	0.008	11.229
手	−1.044	0.471	4.913	0.027	0.352	航	1.671	0.819	4.165	0.041	5.317
什	3.046	0.625	23.733	0.000	21.041	浩	1.116	0.408	7.465	0.006	3.052
今	0.350	0.153	5.219	0.022	1.419	验	1.464	0.711	4.239	0.040	4.322
匀	1.903	0.894	4.530	0.033	6.706	职	1.339	0.516	6.741	0.009	3.814
打	1.505	0.747	4.054	0.044	4.503	彬	1.968	0.962	4.186	0.041	7.154
卉	1.843	0.764	5.816	0.016	6.316	梭	1.990	0.921	4.670	0.031	7.312
功	−0.978	0.507	3.717	0.054	0.376	雪	0.362	0.143	6.426	0.011	1.437
甘	0.707	0.357	3.924	0.048	2.027	悬	1.128	0.266	18.029	0.000	3.091
东	0.463	0.132	12.330	0.000	1.588	鄂	1.685	0.745	5.117	0.024	5.393
田	0.760	0.192	15.686	0.000	2.139	崔	0.701	0.287	5.985	0.014	2.017
叨	1.537	0.662	5.390	0.020	4.652	婴	2.227	0.952	5.473	0.019	9.276
失	0.569	0.306	3.443	0.064	1.766	梨	1.866	0.343	29.650	0.000	6.464
禾	1.203	0.640	3.532	0.060	3.331	笼	−1.951	0.832	5.500	0.019	0.142
外	0.413	0.147	7.910	0.005	1.512	彩	0.575	0.277	4.302	0.038	1.777
立	−1.015	0.428	5.619	0.018	0.362	象	−1.531	0.650	5.548	0.019	0.216
头	−0.690	0.278	6.146	0.013	0.502	猎	1.360	0.453	9.013	0.003	3.895
让	1.390	0.503	7.646	0.006	4.015	麻	1.731	0.452	14.676	0.000	5.644
发	−0.490	0.223	4.819	0.028	0.613	鹿	0.997	0.352	8.038	0.005	2.711
式	2.974	0.878	11.469	0.001	19.574	盖	1.006	0.339	8.796	0.003	2.735
寺	−0.906	0.247	13.440	0.000	0.404	渠	1.272	0.482	6.975	0.008	3.569
西	0.356	0.143	6.207	0.013	1.428	梁	1.405	0.217	42.016	0.000	4.075
百	−0.612	0.295	4.291	0.038	0.542	惟	0.836	0.289	8.361	0.004	2.308
存	0.779	0.360	4.671	0.031	2.179	隋	1.773	0.602	8.673	0.003	5.889
师	−0.882	0.383	5.296	0.021	0.414	塔	1.413	0.475	8.837	0.003	4.107
吐	1.061	0.467	5.159	0.023	2.888	雁	0.581	0.197	8.756	0.003	1.789
舌	1.794	0.744	5.821	0.016	6.013	赏	0.775	0.250	9.622	0.002	2.171
竹	0.657	0.147	19.910	0.000	1.928	赋	0.719	0.220	10.655	0.001	2.051
任	−0.913	0.407	5.035	0.025	0.401	筒	2.140	0.925	5.350	0.021	8.503
后	0.374	0.155	5.827	0.016	1.454	御	0.728	0.211	11.913	0.001	2.071
行	−0.300	0.144	4.369	0.037	0.741	逾	1.444	0.474	9.261	0.002	4.236
旭	1.545	0.838	3.401	0.065	4.689	释	2.004	0.702	8.159	0.004	7.420
冰	−1.149	0.517	4.936	0.026	0.317	然	−0.762	0.267	8.156	0.004	0.467
州	−0.669	0.213	9.844	0.002	0.512	温	−2.620	1.166	5.052	0.025	0.073
池	0.419	0.165	6.486	0.011	1.521	游	0.288	0.140	4.214	0.040	1.334
兴	0.534	0.184	8.432	0.004	1.705	寓	1.274	0.341	13.951	0.000	3.576
寻	0.398	0.186	4.546	0.033	1.488	肆	2.039	0.751	7.369	0.007	7.685
妃	1.266	0.472	7.190	0.007	3.546	献	0.989	0.294	11.343	0.001	2.689
买	1.631	0.384	18.012	0.000	5.111	禁	0.967	0.292	11.006	0.001	2.631
攻	2.662	0.776	11.771	0.001	14.324	零	1.053	0.420	6.293	0.012	2.867
拟	−1.616	0.770	4.402	0.036	0.199	愚	−3.105	1.323	5.510	0.019	0.045
花	0.465	0.111	17.588	0.000	1.592	暗	−0.760	0.325	5.481	0.019	0.468
芳	0.702	0.164	18.324	0.000	2.018	蜀	−1.300	0.562	5.346	0.021	0.272
杏	1.999	0.309	41.801	0.000	7.383	数	−1.083	0.420	6.639	0.010	0.338
轩	0.579	0.248	5.439	0.020	1.785	溪	−0.749	0.265	8.006	0.005	0.473
吴	1.186	0.221	28.724	0.000	3.274	寝	1.667	0.398	17.581	0.000	5.297
里	0.348	0.133	6.854	0.009	1.416	嫁	2.086	0.560	13.888	0.000	8.053
呜	2.024	0.685	8.736	0.003	7.565	熏	1.774	0.676	6.896	0.009	5.895
何	−0.319	0.122	6.840	0.009	0.727	漆	2.899	0.704	16.949	0.000	18.148
邻	0.504	0.231	4.749	0.029	1.655	蔬	1.506	0.411	13.420	0.000	4.508
亩	1.597	0.537	8.854	0.003	4.937	影	−0.499	0.219	5.195	0.023	0.607
弟	0.907	0.273	11.062	0.001	2.477	蝶	0.976	0.292	11.187	0.001	2.653
沉	−1.209	0.609	3.946	0.047	0.298	澜	1.615	0.701	5.305	0.021	5.028
词	0.622	0.233	7.109	0.008	1.863	橘	1.098	0.380	8.342	0.004	2.999
陈	1.113	0.275	16.327	0.000	3.043	繁	0.506	0.277	3.333	0.068	1.658
妨	−18.709	3593.375	0.000	0.996	0.000	爵	1.943	0.897	4.694	0.030	6.978
武	−1.072	0.443	5.868	0.015	0.342	疆	2.155	0.657	10.758	0.001	8.630
坡	2.151	0.631	11.637	0.001	8.597	灌	3.146	0.506	38.629	0.000	23.237
若	−0.868	0.332	6.851	0.009	0.420	扉	1.209	0.252	22.921	0.000	3.349
苔	−1.499	0.399	14.095	0.000	0.223	迥	1.107	0.292	14.341	0.000	3.024
林	0.979	0.118	68.439	0.000	2.661	嗟	0.789	0.360	4.798	0.028	2.200
杯	0.753	0.229	10.807	0.001	2.123	霁	0.975	0.320	9.289	0.002	2.652
事	0.379	0.135	7.931	0.005	1.461	宸	1.439	0.390	13.575	0.000	4.215
岩	−1.284	0.447	8.251	0.004	0.277	皎	1.478	0.462	10.252	0.001	4.384
氛	−2.441	1.228	3.953	0.047	0.087	曛	1.504	0.554	7.378	0.007	4.501
使	−0.943	0.314	8.996	0.003	0.390	岘	−19.481	4936.270	0.000	0.997	0.000
径	0.480	0.215	4.999	0.025	1.617	畦	1.251	0.486	6.636	0.010	3.494
采	−1.784	0.590	9.151	0.002	0.168	骖	1.442	0.529	7.439	0.006	4.228
庞	2.902	0.800	13.169	0.000	18.202	梓	1.629	0.756	4.643	0.031	5.100
郊	1.138	0.231	24.348	0.000	3.121	畿	1.586	0.640	6.133	0.013	4.885
单	1.223	0.558	4.807	0.028	3.396	迸	1.684	0.750	5.047	0.025	5.387
泣	−1.792	0.761	5.538	0.019	0.167	霓	1.322	0.650	4.132	0.042	3.750
宛	1.267	0.445	8.104	0.004	3.551	邺	2.745	0.659	17.353	0.000	15.571
居	0.376	0.154	6.004	0.014	1.457	帏	1.959	0.701	7.817	0.005	7.093
承	0.758	0.273	7.686	0.006	2.134	汴	1.659	0.630	6.926	0.008	5.253
驾	0.753	0.339	4.931	0.026	2.123	蹊	1.497	0.705	4.509	0.034	4.467
春	0.431	0.106	16.520	0.000	1.538	夔	2.288	0.832	7.563	0.006	9.852
挂	0.871	0.333	6.847	0.009	2.388	阊	1.564	0.660	5.607	0.018	4.778
荒	0.492	0.238	4.291	0.038	1.636	羲	1.678	0.853	3.865	0.049	5.354
柳	0.434	0.163	7.053	0.008	1.543	玳	1.420	0.736	3.724	0.054	4.135
柱	−18.454	3766.008	0.000	0.996	0.000	阆	2.504	0.848	8.719	0.003	12.232
树	0.406	0.131	9.675	0.002	1.501	萱	1.710	0.789	4.690	0.030	5.528
砌	−1.749	0.852	4.212	0.040	0.174	荀	1.914	0.719	7.087	0.008	6.782
鸦	0.929	0.452	4.224	0.040	2.532	嵯	1.806	0.785	5.290	0.021	6.085
皆	−0.971	0.426	5.200	0.023	0.379	幄	2.073	0.720	8.289	0.004	7.951
背	−2.479	1.046	5.620	0.018	0.084	罅	2.214	0.855	6.704	0.010	9.148
星	−0.732	0.325	5.076	0.024	0.481	麝	2.916	0.711	16.807	0.000	18.463
虹	−2.564	1.116	5.273	0.022	0.077	帻	3.055	0.740	17.034	0.000	21.217
律	−2.161	1.072	4.062	0.044	0.115	泫	2.546	0.786	10.483	0.001	12.753
阁	0.513	0.224	5.239	0.022	1.670	濠	2.539	0.807	9.892	0.002	12.661
送	−0.387	0.135	8.204	0.004	0.679	**C**	**−4.542**	**0.134**	**1140.940**	**0.000**	**0.011**

The C in the last line refers to Constant.

**Table 2:** Important risk factors (Exp (B) > 3).

Sig⩽0.001			0.001ᐸSig⩽0.01		
Chinese Character	Main Meaning	Exp (B)	Chinese Character	Main Meaning	Exp (B)
灌	Irrigate	23.237	濠	Hao River	12.661
帻	Scarf	21.217	阆	refer to Lang Garden	12.232
麝	Musk deer	18.463	租	Hire	11.229
庞	refer to Pang Degong Garden	18.202	罅	Cracks in a rock	9.148
漆	Lacquer	18.148	幄	Tent	7.951
邺	Ye City	15.571	释	Buddhist	7.420
泫	Crystal-clear	12.753	帏	Curtain	7.093
鸭	Duck	10.581	荀	Xun, a surname	6.782
疆	refer to Gu Pijiang Garden	8.630	熏	Fumigate	5.895
坡	Slope	8.597	隋	refer to Garden of Sui Dynasty	5.889
杏	Apricot	7.383	汴	Bianzhou	5.253
梨	Pear	6.464	给	refer to Jigudu Garden	5.025
麻	Nettle	5.644	亩	Mu, unit of area	4.937
寝	Sleep	5.297	曛	Dim glow of setting sun	4.501
买	Buy	5.111	逾	Exceed	4.236
蔬	Vegetables	4.508	骖	A team of three horses	4.228
皎	Clear and bright	4.384	塔	Buddhist pagoda	4.107
宸	Imperial palace	4.215	猎	Hunt	3.895
梁	refer to Liang Garden	4.075	职	Duty	3.814
寓	Residence	3.576	渠	Canal	3.569
扉	Door leaf	3.349	宛	Winding	3.551
吴	Wu, a place name	3.274	妃	Wife of a prince	3.546
郊	Suburbs	3.121	畦	Farmland	3.494
悬	Hang	3.091	丰	Enrich	3.165
洛	Luo	3.066	浩	Vast	3.052
陈	Mr. Chen	3.043	迥	Far	3.024

Through careful induction, summary and analysis, results of quantitative model have been summarized into the following four aspects, namely regions, ecology, architecture and human behavior, which is also consistent with the literature review. The quantitative model obtained results containing 199 variables. Study conducted text checking on each variable to eliminate some accidental factors. This process can be called coincidence cleaning of the result model. Researchers of this article read and checked the poems of each factor one by one to eliminate the problem of out-of-context quotations, excluding 16 accidental factors from all 145 risk factors. Finally, study summarized four aspects in the trustworthy elements. The following discussion would focus on these four aspects.

## 4 Discussion

### 4.1 Regions

**Fig. 4:**
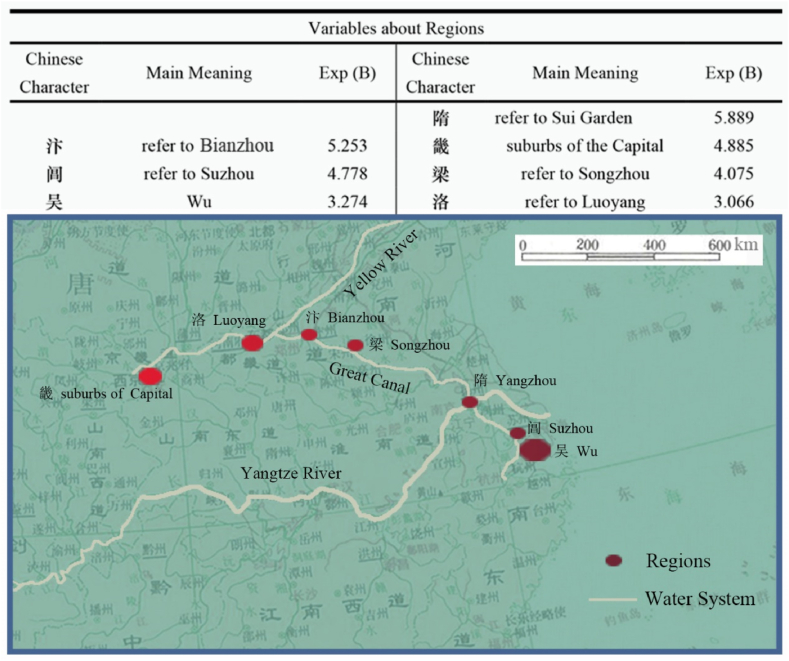
Distribution of regions.

Judging from Fig. 4, gardens in Tang Dynasty were concentrated around the water system formed by the Yellow River, the Grand Canal and the Yangtze River. Among them, four cities appeared along the Grand Canal, namely Bianzhou, Songzhou, Yangzhou and Suzhou. Undoubtedly, in the middle and upper reaches of Yellow River, the suburbs of Chang'an, and Luoyang were also places where gardens flourished. The core area of the newly developed Jiangnan was Wu, where was one of the most beautiful areas in Tang Dynasty, having a large number of private gardens. The research shows the opening of Grand Canal spurred gardening movements along its route. It indirectly confirms the Grand Canal's huge impact on Chinese economy in the Middle Ages [68]. After the Anshi Rebellion, Bianzhou's strategic position as the hub of Great Canal and Yellow River became increasingly important, and its economy became increasingly prosperous. However, direct depictions of the gardens in Bianzhou City were seldom found in Tang poetry; instead, there was a greater emphasis on navigating through Bianzhou by boat and reaching Liang Garden, an expansive garden situated 150 km southeast of it. Liang Garden was originally a huge garden of King Liang in the Western Han Dynasty. The renowned Liang Garden, celebrated for its exquisite landscapes, attracted numerous poets who sought inspiration from the Han Dynasty monuments and towering ancient trees it housed. Situated on the outskirts of Songzhou actually, this great garden became an integral part of the scenic spots mentioned in Tang poems about Bianzhou and Songzhou. According to Sima Qian's records, Liang Garden spans over 50 square kilometers, showcasing a remarkable scale that offers a glimpse into the magnificence of pre-Tang Dynasty gardens. 500 km southeast from Songzhou, Yangzhou was an important node of the Grand Canal. In this prosperous water city, the expansive imperial gardens commissioned by Emperor Yang during the Sui Dynasty had endured into Tang Dynasty. However, there were more private gardens here. During the Tang Dynasty, it was widely acknowledged that "Yangzhou ranked first in terms of prosperity, followed by Yizhou.” Yangzhou was renowned as a garden city surrounded by water, where boats were more prevalent than carts and horses. The frequency of passing boats created a lively atmosphere in the neighborhoods [42]. About 200 km southeast along the canal from Yangzhou was the historic city of Suzhou. Its most famous attraction should be the Wu Garden ruins where King Wu went hunting during the Spring and Autumn Period. In addition, Suzhou City in Tang Dynasty had been dotted with a large number of official gardens and private gardens, all of which were connected to canals through the city's water system [1]. Since Spring and Autumn Period, Suzhou City had been the center of Wu, and in the wider Wu area, people had always been fond of creating gardens suitable for life among green mountains and clear waters. In brief, the route from Chang'an along the majestic Yellow River to Bianzhou, and then from Bianzhou along the magnificent Grand Canal to Wu, unveiled a pathway where gardens thrived during the illustrious Tang Dynasty.

### 4.2 Ecology

**Fig. 5:**
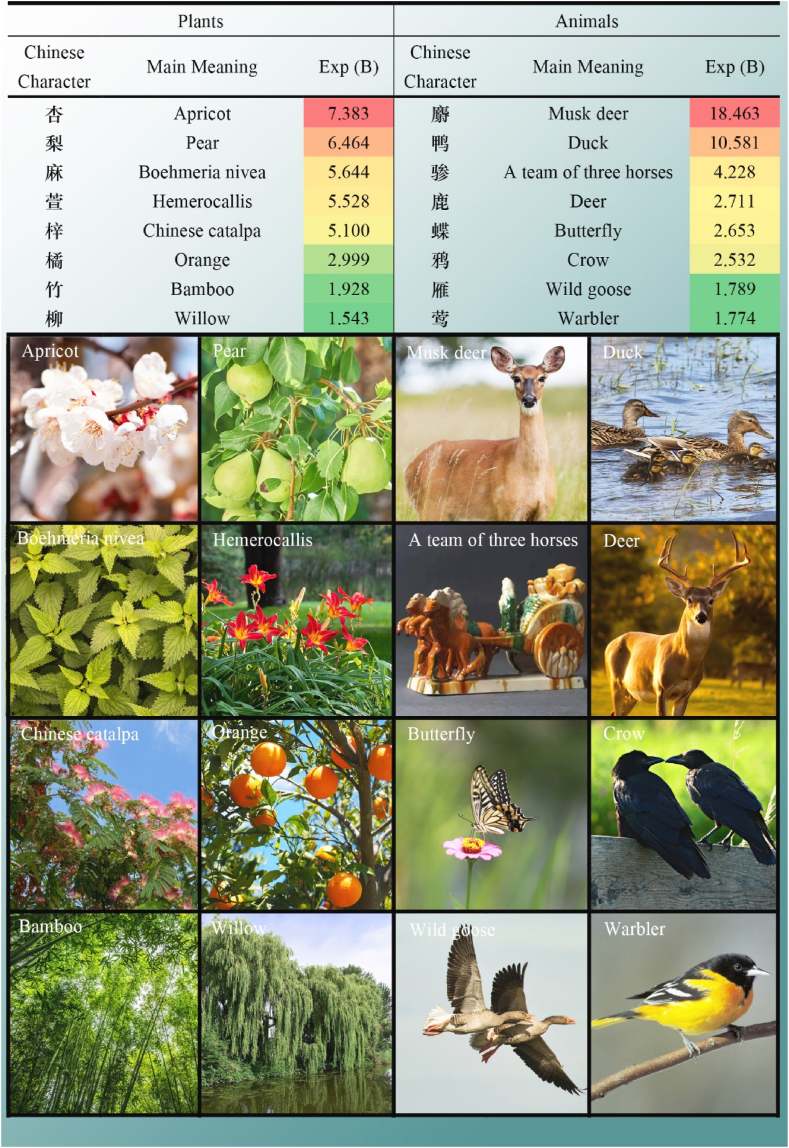
Specific plants and animals.

**Fig. 6:**
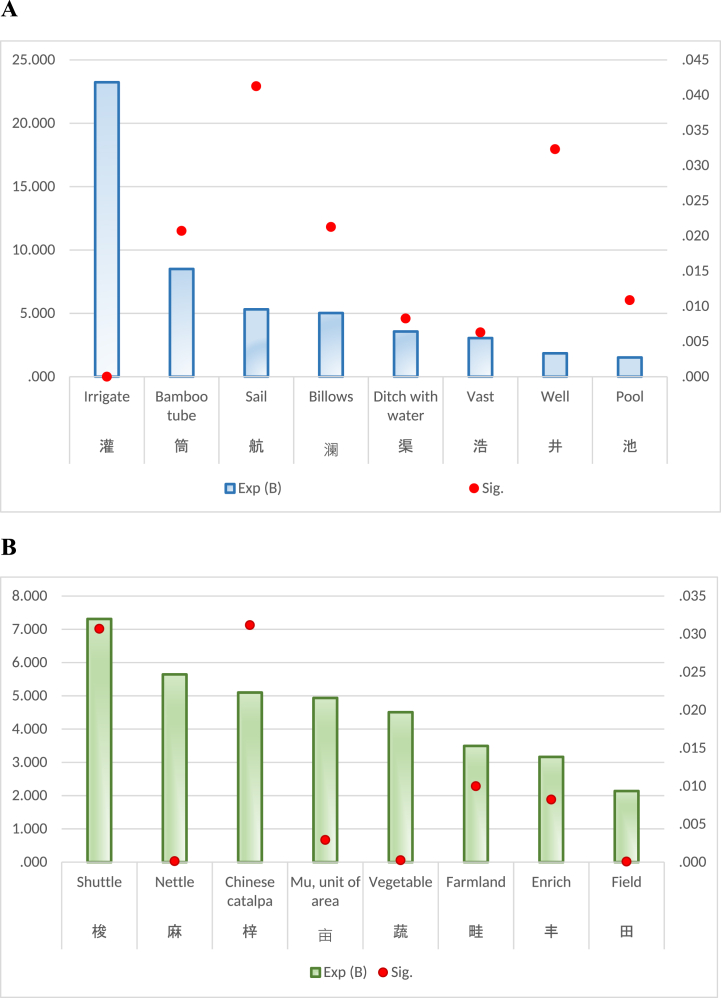
A Water system. **B** Agriculture and textiles.

Through the exploration of garden ecology, it has been recognized that garden of Tang Dynasty was an ecosystem that attached equal importance to production, life and appreciation. It is consistent with the literature mining research of Yuxi Zhou et al. [69]. Study of ecological aspects in Tang gardens has many surprising findings, such as the animals and plants around gardens, the water system of gardens, as well as the agriculture and textile industry in the gardens. Common plants in Tang gardens are presented in Table 3, while Fig. 5 illustrates the specific flora and fauna surrounding these gardens. Additionally, Fig. 6A showcases the elements of water system, whereas Fig. 5B highlights the agriculture and textile industries associated with Tang Dynasty gardens.

**Table 3:** Common plants.

Chinese Character	Main Meaning	Sig.	Exp (B)
卉	Grass or flower	0.016	6.316
蔬	Vegetables	0.000	4.508
林	Grove	0.000	2.661
花	Flower	0.000	1.592
树	Tree	0.002	1.501

A large number of Tang Dynasty gardens were filled with productive plants and animals. As shown in Table 3, Tang Dynasty gardens were filled with flowers, grasses, vegetables, groves and trees. As shown in Fig. 4, the most common ones in Tang Dynasty gardens were apricot, pear, *Boehmeria nivea*, *Hemerocallis*, Chinese catalpa, orange, bamboo and willow. Among them, trees of apricot, pear and orange have both ornamental and edible value. *Boehmeria nivea*, Chinese catalpa and bamboo were frequently used for production. The large-scale planting of bamboo is consistent with the research results of Peiyao Hao and Li Dong [70]. Fiber of *Boehmeria nivea* could be used to make clothes, catalpa could be used to make all kinds of furniture, while bamboo could be used to make various utensils. Plants that were popular in Tang gardens also included *Hemerocallis* and willows, which were generally considered to be beautiful ornamental plants. At the same time, it was not unusual for people in the Tang Dynasty to raise ducks in their gardens. Strangely, deer, or musk deer were often kept in gardens, which have both ornamental and other uses. For example, Tang people believed that deer antlers and musk could be used for traditional Chinese medicine. In addition, the animals that often appeared in and around Tang Dynasty gardens were butterflies, crows, wild geese and warblers. The frequent appearance of Can 骖 in gardens suggests that the owners or visitors of gardens in the Tang Dynasty were people of high social status or wealthy people. Because it means a carriage composed of three horses.

Tang Gardens had innovative and intricate water systems within their gardens, which served multiple purposes. Apart from providing a picturesque setting for leisurely contemplation, these elaborate water systems catered to the agricultural and textile needs of the Tang people. Fig. 5A reveals that irrigation played a predominant role in these gardens. The presence of wells, pools, ditches, and bamboo tubes further emphasized the comprehensive nature of the irrigation system. Such configuration is consistent with Jing Qiu's research on the water systems of Tang Dynasty gardens [71]. It is fascinating to imagine people sailing through these vast gardens during the Tang Dynasty, creating sparkling billows on the water as they navigated through this ingenious network. Moreover, agriculture held great importance in Tang gardens. The cultivation of nettles, catalpas, and vegetables showcased their meticulous farming practices. Nouns such as "mu” (a unit of land measurement), "field,” and "farmland” indicate that agriculture was an integral part of their daily lives. These gardens not only provided aesthetic pleasure but also contributed to sustaining their agricultural endeavors. Additionally, Fig. 6B suggests that textile industry thrived within Tang Dynasty gardens. Nettle plants were a primary raw material used in textile production during this period [72]. The presence of shuttles as essential tools further supports the notion that textiles were produced extensively within these garden spaces. This integration of textile industry highlights how multifaceted and self-sufficient these gardens were in meeting various societal needs. The aforementioned study unveils an expansive and resplendent garden ecology during the Tang Dynasty, which exuded both breathtaking beauty and remarkable practicality.

### 4.3 Architecture

**Fig. 7:**
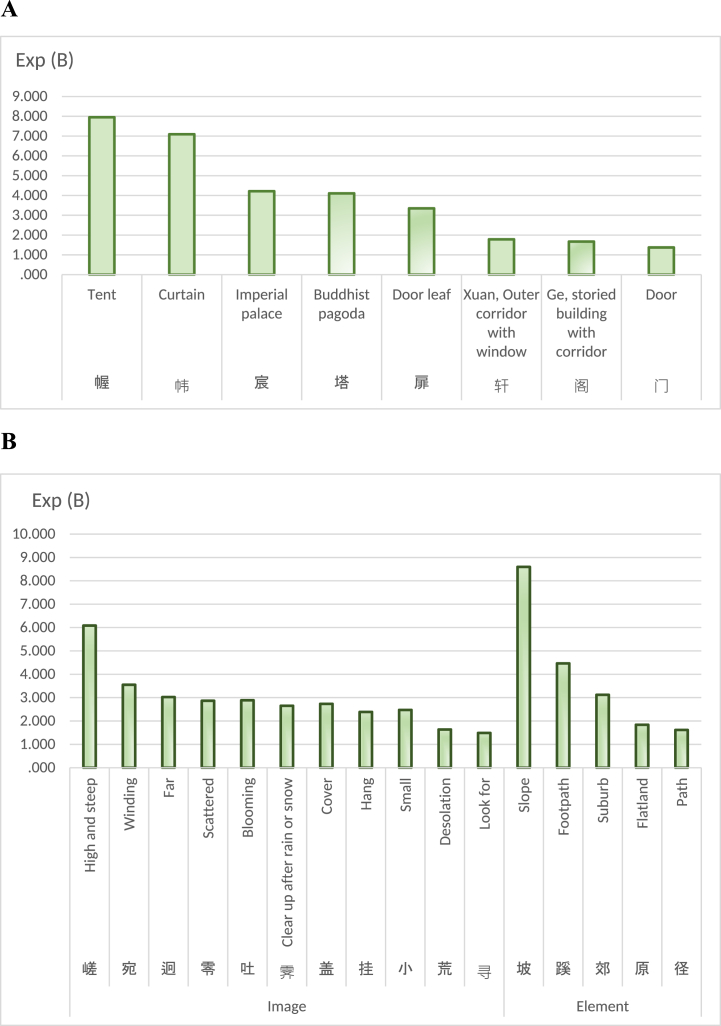
A Architecture & component. **B** Image and element of space.

According to the study, architecture of Tang gardens possesses its own distinctiveness of nature and simple, while also exhibiting numerous features that were subsequently inherited by the gardens of the Ming and Qing Dynasties. Garden architecture of Tang Dynasty indeed exhibited the features of its era, showcasing unique building types such as tents and pagodas. It also featured distinctive spatial elements and imagery, displaying significant variations in height, vast expanses, winding pathways, and an abundance of flowers and trees. All these aspects reflect the simplicity of nature with minimal artificial intervention. This assessment aligns with Zhang and Lian's research on Tang Dynasty gardens [1], as well as the research of Menghan Bao et al. [73]. From Fig. 7A, appearance of tents and curtain in Tang Dynasty gardens was unique. The reason why the Tang people were keen on setting up tents in gardens was related to the fact that the Tang nobles had some nomadic ancestry. Although they were in the core area of agricultural civilization, they still retained some Hu style. Next, appearance of pagodas in gardens also benefited from the great popularity of Buddhism in the Tang Dynasty. As well known, the Tang Dynasty was the most prosperous era of Buddhism. The nobles had the tradition of "turning their houses into temples”, and their houses were gardens frequently. In addition, study has observed the emergence of Xuan and Ge. These two forms of architecture often appeared in gardens of Ming and Qing Dynasties, which shows that a large number of architectural forms in Tang gardens were indeed inherited by the gardens of Ming and Qing. Signally, curtains, doors, and leaves often appear in Tang gardens, indicating that the multi-layered sense of Tang Dynasty gardens had been formed. This spatial feature is not exclusive to Ming and Qing gardens. Judging from Fig. 7B, collection of spatial elements suggests that Tang gardens often appeared in plain areas in suburbs, and gardens often have slopes and various paths. When people got into a garden of Tang Dynasty, the spatial imagery was truly remarkable. Variations in height created a visually stunning landscape, with towering peaks and deep valleys that added depth to the overall scenery. As one explored further, he would come across vast expanses of open spaces, providing a sense of freedom and tranquility. Winding pathways within this environment were not only aesthetically pleasing but also served as an invitation for exploration. These pathways meandered through lush greenery, leading visitor on a journey filled with anticipation. Moreover, the lush flowers, plants and trees added bright colors to the surrounding environment, and visitor would see scattered flowers and leaves on the ground. Overall impression conveyed by this image is one of inherent elegance and effortless simplicity, yet brimming with wild interesting.

### 4.4 Human behavior

**Fig. 8:**
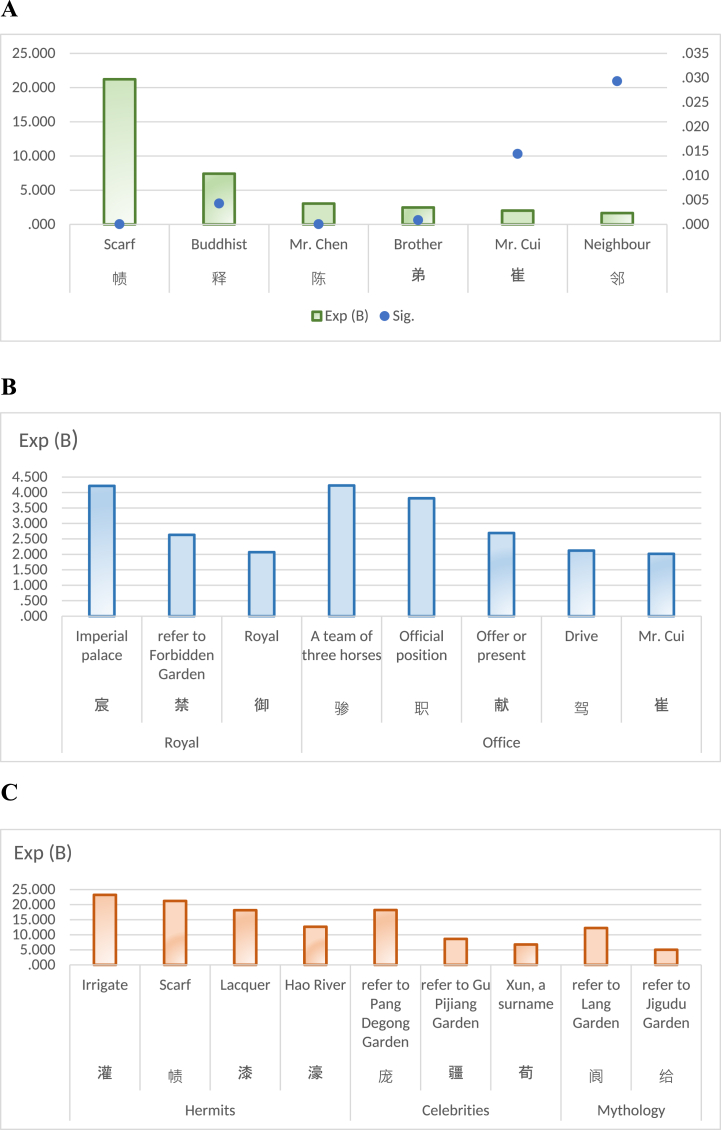
A People around garden. **B.** Royal and official **C.** Allusions: hermits, celebrities and immortals.

Gardens were favored by royal family, nobility, officials, literati, and monks alike, according to the study: on a spiritual level, they sought solitude like hermits, fame like celebrities or lifestyle like immortal; however, on a practical level, their pursuit was centered around finding pleasure through activities such as socializing over drinks and conversations, engaging in hunting expeditions or games, composing poetry and indulging in restful slumber; what's more, they tried to experience full spectrum of life's beauty encompassing visual splendor, fragrant aromas, exquisite flavors and emotional release. Depiction in Fig. 8 showcases individuals associated with gardens during Tang Dynasty. Among them, Fig. 8A provides a concise overview of specific individuals, Fig. 8B presents an encompassing portrayal of various royals and officials, while Fig. 8C summarizes the characters found in diverse allusions. Scarf, as depicted in Fig. 8A, was a prevalent attire among ancient literati, often symbolizing the identity of a hermit. Additionally, presence of Buddhists reflects the widespread influence of Buddhism during Tang Dynasty. Furthermore, it is unsurprising to find Mr. Chen and Mr. Cui frequently appearing in garden scenes since both surnames held significant prominence during this era. Cui being ranked first among the five most renowned aristocratic surnames [74], as well as Chen had numerous influential individuals. For example, the poet Qian Qi wrote a poem “Feast for Fuma (emperor's son-in-law) Cui at his Villa in Yushan” to commemorate the grand occasion of drinking in the garden of Fuma Cui, who was a descendant of the noble Boling Cui family. Another famous poet Li Shangyin wrote two poems "Chen Hougong” on the site of Mao Garden to commemorate the luxurious life of the Chen Dynasty emperor in the harem. In Fig. 8B, it showed various royal and official elements, indicating that gardens were indeed places associated with the powerful and elite. Judging from Fig. 8C, it is intriguing to note that gardens were intricately intertwined with various characters in numerous allusions. For instance, act of donning a scarf while irrigating to farmland often symbolizes the solitary existence of a hermit, whereas clerk at Qi Garden and debate held at Hao River both referring to Zhuangzi, who epitomizes Chinese hermit culture. Next, Pang Degong, Gu Pijiang, and Xun Yu were virtuous celebrities in history who lived in seclusion of countryside gardens. This explains ideal life of traditional Chinese, which should be a free and virtuous person. Lastly, Lang Garden embodied the ethereal realm of fairy Garden, while Jigudu Garden represented the serene atmosphere of Buddhist gardens. Both types of gardens hold high significance as cultural symbols in traditional China. Undoubtedly, gardens had served as pivotal places for Buddhism and Taoism until Tang Dynasty, since many Buddhist and Taoist temples were located in gardens.

**Fig. 9:**
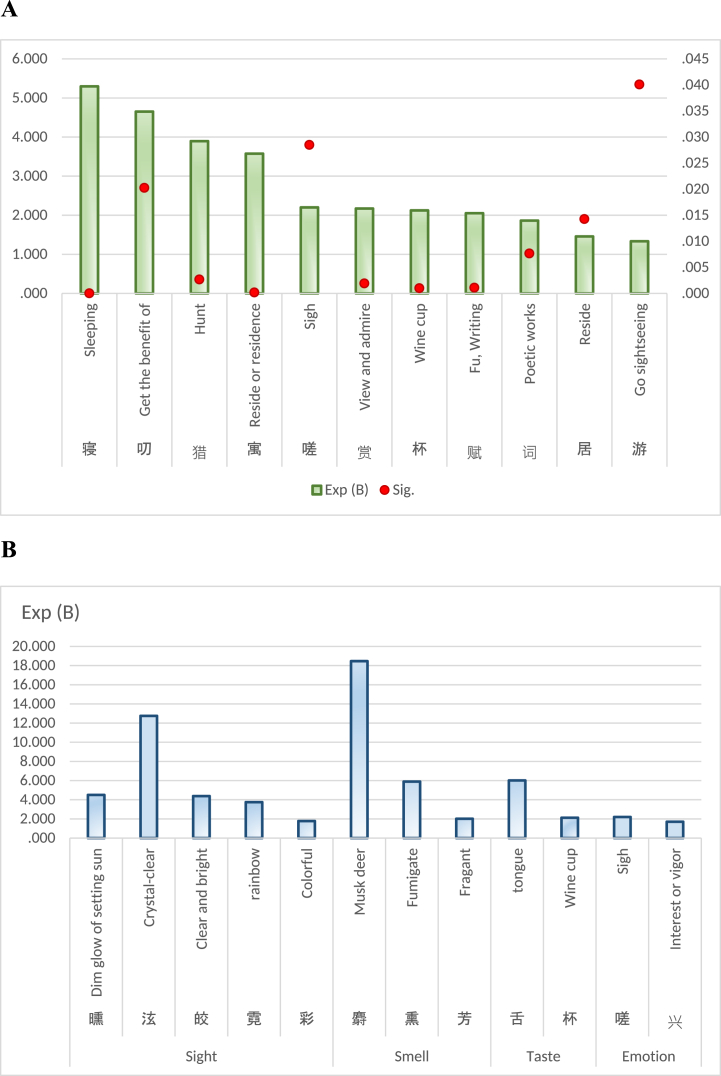
A Behavior. **B** Sense & emotion.

In addition to the pursuit of pure spiritual realm, people in Tang Dynasty also sought solace or enjoyment in the beauty of garden nature. Gardens served as a place where they could escape from the pressures of everyday life. They created places that people could immerse themselves in a carefree or comfortable existence. As seen from Fig. 9, individuals would gather with friends to socialize over drinks. They engaged conversations within gardens, which provided an ideal setting for fostering social connections. Furthermore, hunting expeditions were popular pastimes that allowed them to showcase their skills while enjoying friendly competition. It also proves that the scale of Tang Dynasty gardens was much larger and rougher than that of Ming and Qing Dynasty gardens. As expected, poetry held great significance during this era, with many individuals composing verses inspired by their surroundings. The serene ambiance offered inspiration for creative expression, resulting in beautiful poems. It had been proven instrumental in capturing both personal sentiments and meticulous observations of nature's splendor. Pursuit of pleasure extended beyond socializing and recreational activities; it encompassed experiencing all aspects of life's beauty within gardens. People delighted in visual splendor through meticulously designed environment adorned with vibrant flowers, meandering streams, morning dew and evening sunset. The air was filled with fragrant aromas emanating from blooming flowers or incense burners placed throughout the garden. With wines and cups, exquisite flavors tantalized taste buds as elaborate feasts were prepared using fresh ingredients sourced directly from nearby fields or fish ponds within the garden itself. This moment had been witnessing a remarkable surge in everyone's interest, as people reveled in the pure interesting of unabashedly unleashing their emotions.

### 4.5 A comprehensive case: Ludaofang Garden of Bai Juyi

**Fig. 10:**
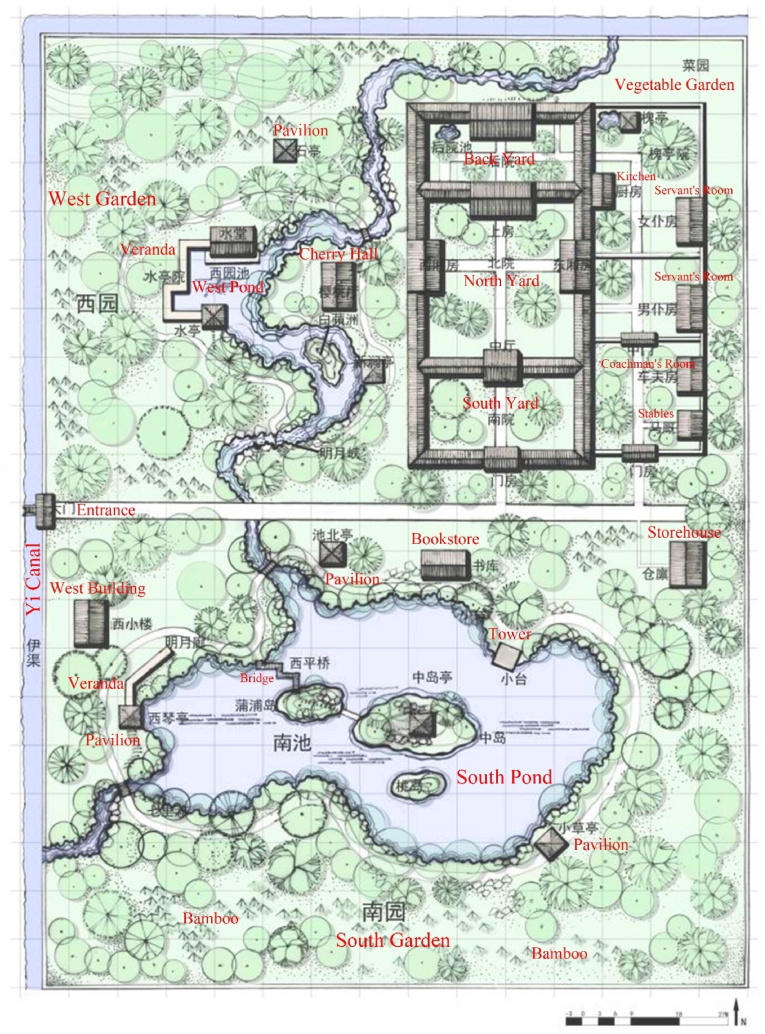
Site plan of Ludaofang Garden. Editing based on Jing Qiu's original manuscript [71].

To understand the Tang gardens intuitively, study would take Bai Juyi's Ludaofang Garden as a case. Bai Juyi was one of the most famous poets in Tang Dynasty, and this garden was his most important garden. He lived here for a long time in his later years. This garden was located in Luoyang, where many retired senior officials of the Tang Dynasty lived in Ref. [20]. The location of Ludaofang was very suitable for garden-like life because it was located in the southeast side of the city, which was a relatively quiet area fulling of gardens with flourishing flowers and trees. Overall, it was a typical garden of Tang Dynasty. The study would analyze it from four aspects: regions, ecology, architecture and human behavior. From a regional aspect, Luoyang was the second capital of the Tang Dynasty. Located in the middle reaches of the Yellow River, it was one of the most economically and culturally prosperous cities in the Tang Dynasty [8]. From an ecological perspective, Ludaofang Garden was a garden with lush flowers and trees. The Yi Canal flowed through here, allowing the garden to easily connect to the water system [19]. Therefore, this garden had covered an area of nearly 2.23 acres, of which the water area accounts for 1/5 [71]. As seen from Fig. 10, this water system had the function of irrigation and was also suitable for boating. There was a vegetable garden on the northeast of the garden, indicating that the owner used the garden for some production. According to the poet's records, the entire garden was planted with bamboos, many locust trees and willow trees were planted along the water edge, and the pond was full of lotus flowers. Obviously, west side of the Cherry Hall was planted with cherries [24]. By the way, the owner also raises two Huating cranes. From an architectural perspective, the residence was arranged on the northeast side, with larger gardens on the west and south sides. This layout was very similar to the Japanese shinden garden. Unsurprisingly, ancient architecture of Japan was greatly influenced by Tang Dynasty architecture. On the east side of the main courtyard, the servants' room, coachman's room and stables formed the auxiliary courtyard. From the plan, one can saw the storehouse, bookstore and Qin (a string instrument) pavilion, indicating that the owner's elegant hobbies were integrated with real life. The garden had at least 6 pavilions and multiple verandas, indicating that the gray space connecting the internal and external spaces was crucial in the garden. In his daily retirement life, the poet immersed himself in the garden, playing the Qin (a string instrument), drinking, and admiring Taihu stones. In fact, Bai Juyi also brewed wine himself. As a Buddhist, he also drank tea in the garden besides religious activities. Drinking tea was a very ritualistic thing in the Tang Dynasty, which required a complete set of procedures and utensils. Of course, as a scholar, he had a huge bookstore that he could read at any time. As a poet, he wrote poems in the garden every day, describing his optimistic lifestyle. As a retired official, Bai Juyi was still keen on socializing. The most famous event was that he organized a drinking party for nine retired officials in his garden [75].

## 5 Conclusion

This paper restores the feature framework of Tang gardens from four aspects: regions, ecology, architecture and human behavior. Tang gardens were primarily located in close proximity to the waterways created by the Yellow River, Grand Canal, and Yangtze River. Secondly, Tang gardens functioned as a balanced ecosystem, encompassing production, livelihood, and aesthetic appreciation. They were renowned for their innovative and intricate water systems, which served multiple purposes including agricultural and textile needs, in addition to providing a picturesque setting filled with productive plants and animals. Next, architecture of Tang gardens showcases its unique natural and simplistic qualities, while also displaying various features that were later passed down to the gardens of Ming and Qing Dynasties. Lastly, Tang gardens were highly favored by royalty, nobility, officials, intellectuals, and religious practitioners. On spiritual level, they sought to be hermits, immortals or practitioners far from the world; however, on a practical level, they actively engaged in secular activities, while also indulging in recreational pursuits to enhance their sensory experiences in aesthetic elegance.

## 6 Limitations and future research

Firstly, logistic regression is a research method suitable for correlation research rather than causal research. This makes it only ideal for initial stage of exploratory research. Secondly, samples used in this study did not encompass Complete Tang Poems. Actually, study focused on five-character and eight-line poems, totaling 13,100 poems which represent approximately one-fourth of the entire collection of Tang poetry. In future investigations, it would be beneficial to gather samples from other types of Tang poetry such as five-character & four-line or seven-character & four-line compositions. They could be used to conduct specific studies or comparative studies.

## Data availability statement

The data associated with the study have not been deposited in a publicly available repository. Data will be made available on request.

## CRediT authorship contribution statement

**Biyu Huang:** Funding acquisition, Formal analysis, Data curation, Conceptualization. **Zhenwei Zhang:** Project administration, Methodology, Investigation. **Wende Chen:** Writing – review & editing, Writing – original draft, Visualization, Validation, Supervision, Software, Resources.

## Declaration of competing interest

The authors declare that they have no conflict of interest whatsoever with regard to the doing and publishing of this research.
